# Considerations for Designing Context-Aware Mobile Apps for Mental Health Interventions

**DOI:** 10.3390/ijerph16071197

**Published:** 2019-04-03

**Authors:** Ignacio Miralles, Carlos Granell

**Affiliations:** Geospatial Technologies Research Group (GEOTEC), Universitat Jaume I, Av. Vicente Sos Baynat s/n, 12071 Castellón de la Plana, Spain; mirallei@uji.es

**Keywords:** geographic concepts, place, sense of place, mental health interventions, m-health, gamification, context-aware mobile apps, location-based interfaces, design considerations

## Abstract

This work identifies major areas of knowledge and proposes a set of relevant dimensions by area that must be taken into account in the design and delivery of context-aware mobile applications for mental health interventions. We argue that much of the related research has focused only on a few dimensions, paying little or no attention to others and, most importantly, to potential relationships between them. Our belief is that the improvement of the effectiveness of mobile interventions to support mental health necessarily implies that developers and therapists comprehensively consider the interaction between the proposed dimensions. Taking as a starting point the three areas of knowledge (Technology, Context, and Mental Health), we re-examine each area to identify relevant dimensions, discuss the relationships between them and finally draw a series of considerations. The resulting considerations can help therapists and developers to devise, design, and generate custom mobile applications in a way that increases the motivation and engagement of patients and, therefore, the effectiveness of psychological treatments.

## 1. Introduction

Recent data reveal the true extent of mental health disorders. According to a 2014 WHO systematic review [1], one quarter (27%), around 83 million people, of the adult population (aged 18–65) in European Union countries had experienced at least one mental health disorder (e.g., substance use, psychoses, depression, anxiety, eating disorders) in the past year. Other sources cite 165 million Europeans each year suffer a mental health disorder, and more than 50% of the population in middle- and high-income countries suffer from at least one mental disorder at some point in their lives [2]. Within the spectrum of mental health disorders, depression is most prevalent, affecting almost 5% of the world’s population according to 2017 WHO data [3]. A similar number also suffers from a range of anxiety disorders, either separately or as a comorbidity. If we look at the trend, the total estimated number of people living with depression increased by almost 20% between 2005 and 2015 [4].

Furthermore, there are several indications that mental health disorders can expand in a worrisome way, especially in adolescents. San Diego State University Jean M. Twenge, a professor of psychology who has been studying the evolution of American adolescents for 25 years, described the sudden jumps in mental health disorders since 2012, in comparison to the gentle slopes in past epochs [5]. These findings are also supported by a recent international study investigating the prevalence of psychological disorders in eight developed countries among first-year university-level students: one in three students report symptoms of a mental health disorder [6]. All these findings and indicators suggest an increase in the prevalence of mental health disorders in adolescents and young adults in the immediate future. Among other actions, research and innovative developments to alleviate these problems are required to improve public health and reduce the associated socioeconomic impact of the increasing level of mental health disorders [7].

Human health, including mental health, *“is part of an interdependent multifaceted system”* ([8], p. 1023). This is not a new idea. Indeed, other influential factors have been taken into account in the prevalence of mental health issues. For example, environmental health researchers have long recognized the importance of geographic context for understanding the effects of different environmental factors such as noise level and air pollution on mental health [9,10]. Urban practitioners and planners have also studied how the configuration and features of the built environment along with the morphology of cities may influence citizens’ health [11,12,13,14]. Essentially, a large body of research has focused on responding from different perspectives regarding how the natural and built environment affect people’s health, i.e., to understand the degree and ways in which the features and manifestations of the environment influence public health and aspects of our lifestyle in cities [15,16]. Computer scientists are recently gaining importance in the field as the increasing democratization and deployment of mobile computing, mobile devices and sensing technologies [17] provide new opportunities to deliver mobile mental health care [18,19,20] thus improving quality of life [21]. Various authors [20,22,23] have suggested that interventions with mobile phones designed to support evidence-based treatments could even reduce barriers to access and increase participation in treatment. The rapid and wide adoption of these new developments and technological innovations is leading to a new stream of research that explores the prevention of mental health disorders through mobile-based interventions [24].

We recognize the value and vital contributions of past research to the discipline. However, these approaches have taken only a partial and incomplete view of the problem, with little concern to other interrelated dimensions that are crucial parts of the problem. Our belief is that most studies consider a limited view, usually restricted to considering pairs of dimensions such as the built environment and mental health, or mobile technologies and mental health. Ferrás et al. [25] move forwards and add the geography and location to the equation, starting a dialogue about the potential benefits of bringing geography (and location), mobile technology and mental health together [25]. In this context, recent research works confirm the assumption that considering distinct aspects may lead to better tools for mental health interventions. For example, Palmius et al. [26] collect location data from mobile phones and process it to assess the level and regularity of geographic movement of patients. By adding the location in their approach for delivering interventions, their results suggest a strong correlation between geographic movements and patients with depression in bipolar disorder. Saeb et al. [27] follow the same strategy of passively collecting location data from mobile phones to estimate an individual’s most visited places and locations. In a subsequent study, the same authors demonstrate that location data may be a reliable predictor of depressive symptom severity [28].

These examples suggest that a greater variability of the dimensions and factors involved in mental health interventions can have a positive effect in the design and impact of location-based mobile tools for such interventions. We believe that this is a promising step to raise mobile-based interventions to a new level, as taking a holistic view to study the interdependent and multifaceted field of mental health leads to greater levels of contextualization for personalized interventions. This paper expands this idea by conducting an exploratory exercise in the variability of influential dimensions and their interrelations for context-aware mobile apps in mental health interventions. Our long-term research goal is the design and development of a software platform to inform psychologists and therapists for the rapid design and creation of mobile apps that are well suited to mental health disorders. This is aligned with a recent call to implement improved interventions using technological advances (among which include mobile interventions), which was identified as one of the six research priorities in mental health for Europe [7].

This paper’s contribution can be regarded as an important step in the evolution of mobile apps to support mental e-health and wellbeing. In particular, we perform a preliminary analysis to identify interrelated dimensions which could be regarded as conceptual building blocks, similar to the set of software patterns devised by Buschmann et al. [29] and Gamma et al. [30]. In these seminal works, the concept of *software pattern* refers to a generic solution to a specific class of design issues, i.e., a well-defined strategy is applied to solve similar problems. Consider for example, a software tool that is aimed at searching content from multiple social media services (e.g., Twitter, YouTube, Wikipedia, Open Street Maps), alleviating the user from understanding the specific data formats and data access protocols to query and retrieve data from each service. This issue is not new but has been long studied from the perspective of software engineering; Buschmann et al. [29] proposed the software pattern *Broker* that can be easily understood as an intermediate component (middle-ware) between users and the different systems. Users only interact with this middle-ware component instead of with an arbitrary number of distinct systems. Many examples have adopted this pattern in varied application domains [31,32] as a generic solution to reduce complexity and improve scalability and interoperability.

This paper reflects on the foundation for the establishment of *patterns* –i.e., the identification of well-defined strategies, each applicable to diverse scenarios and situations–, for the design of mobile applications for mental health interventions. Our intent is that the discussion of the key dimensions and their relationships allows health professionals to conceive and design better mobile apps in the realm of mental health interventions that fully take advantage of the potentialities and synergies of the three areas of knowledge, namely Technology, Context, and Mental Health. The following research questions (RQs) drive the remainder of this article:

- RQ1: What are the relevant dimensions that sustain or describe each area of knowledge (Technology, Context, and Mental Health)?
- RQ2: How do the dimensions that belong to different areas of knowledge relate to each other?
- RQ3: Which interrelated dimensions may best inform users for designing context-aware mobile apps to support mental health interventions?

## 2. Methods

The present study combines various qualitative research techniques together, following the tenets of the grounded theory method [33,34]. In short, the grounded theory is *“the attempt to derive theories from an analysis of the patterns, themes, and common categories discovered in observational data”* ([35], p. 307). In this study, the “discovered theory” in terms of expected findings is comparable to the set of considerations to inform designers and therapists in the process of designing context-aware mobile applications for mental health interventions. Our choice of research methods to obtain this knowledge from empirical data looks directly at the general theory of scientific knowledge [36], which is composed of facts, concepts, principles, theories, and laws. Our focus in this article is limited to the chain “facts-concepts-principles”, which allows us to move from the observational data (facts) to the establishment of meaningful relationships among facts and concepts (principles).

**Figure 1:**
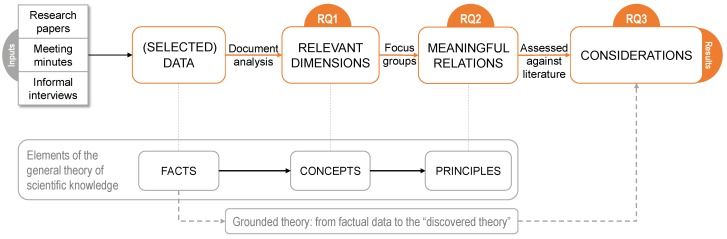
Qualitative research methods in relation to the RQs and their correspondence with the elements of the general theory of scientific knowledge [36] and the basics of the grounded theory method [33,34].

Figure 1 illustrates the qualitative methods approach connected to the RQs (Section 1), and how they correspond with the tenets of the general theory of scientific knowledge and the grounded theory. The extraction of concepts (i.e., significant dimensions) from facts is related to RQ1 (Section 3.2), while the identification of principles (i.e., meaningful relations between dimensions) and their assessment against existing literature refer to RQ2 (Section 3.3) and RQ3 (Section 4), respectively. The combination of various qualitative research methods for the collection and selection of observational data allows us to gather a good understanding and a practical reality of the potential use, benefits and impact of mobile applications for mental health interventions.

To answer RQ1, we used the document analysis method as a qualitative research method. Bowen [37] defines it as *“a systematic procedure for reviewing or evaluating documents”* ([37], p. 27), to find, select, make sense of and synthesize data in the analyzed documents. Extracted data are then *“organized into major themes, categories and case examples”* ([37], p. 28). The advantages of the document analysis in our exploration are two-fold. First, it allowed us to corroborate observational data across sources to reduce the potential bias of using a single information source or research method for data collection [38,39]. We used various sources to obtain a more complete picture of the research topic, which allowed us to validate similar concepts from different sources to triangulate the data findings. Second, as document analysis is a process of evaluating and interpreting the selected documents (e.g., research papers, meeting minutes), content analysis [35] is commonly used to organize key data and information extracted from the documents into categories (i.e., coding) for subsequent data processing and re-examination. This is the main reason behind the identification of relevant dimensions (RQ1) and the re-interpretation of the relations between them (RQ2).

Evaluated documents in the document analysis method are not restricted to published research papers (journal articles and conference papers), but other forms of documents are also relevant, such as books, doctoral theses, minutes of meetings, institutional reports, and other public documents and records. Therefore, document analysis is not a research method for data collection, but it pursues data selection [37]. The selected documents are interpreted to make sense of a particular research topic. Therefore, it is worth noting that document analysis can be used in combination with other quantitative research methods that focus on data collection such as systematic reviews, but is not a type of review. In this study, we did not perform a review. Rather, we use document analysis as a qualitative research method to evaluate a range of selected documents to extract and interpret a set of design considerations. Next, we describe the procedure for selecting the main forms of documents.

Research papers were an important source of documents in our exploration. We selected widely used citation databases such as Scopus and Web of Science. We first compiled a list of key terms for each area of knowledge by screening previous review papers and concept papers from the related literature. Based on internal discussion among the authors and the exchange of information with psychologists through regular meetings (below), we selected the final set of research papers (listed below) as input for the document analysis method.

Regular meetings between the authors (computer scientists and technologists) and a group of psychologists from the LABPSITEC, a research group specialized in applying technology to mental health disorders, were a pragmatic way to establish a continuous dialogue and an effective communication channel between the two disciplines. Informal interviews were also conducted at the same time as the meetings. Nevertheless, only some meetings were followed with interviews of individual therapists. The interviews combined an “interview guided approach” and a “standardized open-ended interview” ([40], pp. 438–442), which means that a few questions were previously predefined according to the planned agenda of the meeting, but the remainder of the questions were based on the content of the conversation. In general, the meetings and informal interviews allowed us to collect essential or distinctive features that would otherwise not be able to be collected via other traditional means such as online surveys and questionnaires. For example, barriers, problems, and fears perceived by therapists of using mobile technology in treatments for depressive symptoms. Equally important was to capture, through these interviews, the expectations of the therapists and future ideas about the application of mobile technology in their mental health interventions [41].

In summary, we interpret and evaluate various forms of documents through the document analysis method, such as research papers, meeting minutes, and observational and qualitative data collected from informal interviews. These are the main types of documents selected for the document analysis method (Figure 1).

To answer RQ2, we ran two focus groups [35]. The first focus group was aimed at exploring the relationship between context and mental health. Given the multidisciplinary objective of the focus group, the background of the participants was intentionally related to distinct disciplines and areas of knowledge such as Context, Mental Health, Education, and Technology. The second focus group was aimed at drawing conclusions about the use of technology in mental health interventions. In this case, the required profiles of participants were more restricted than in the first focus group: health professionals with a certain level of technological expertise was the dominant profile. LABPSITEC researchers helped us with the recruitment of eligible participants for the focus groups.

The final participants of the focus groups were emailed to briefly explain the topics to be discussed ahead of the meeting. On the same day of the meeting they all signed an informed consent form to ensure ethical and privacy considerations of the participants. Both focus groups were recorded. We took notes during the focus group, complemented afterwards by a qualitative analysis and content analysis of the recorded video to extract the main highlights of the meeting. The two focus groups were organized in two phases. The first phase consisted of a guided discussion where participants were asked key questions and could comment and discuss each of them for 20 min. In the second phase, participants could extend the topic and debate more freely with a longer time frame duration. In both focus groups, participants were encouraged to consider the role of geographic elements and location as drivers of the discussion.

As regards the first focus group, centered on the exploration between context and mental health, the key questions were: What and how the characteristics of the urban environment can influence the physical activity/mental health; What aspects of games and how they can be regarded as tools for motivation and engagement; How these games can take advantage of the characteristics of the urban environment. For the second focus group, which was more specialized in the scope than the first, the main theme was the application of technology in mental health interventions. Participants were asked to describe the role of technology in the interventions they have designed and/or actively participated in, as well as to imagine the potential uses of technology that could help answer the following questions. In the case of depression, for example, how many times has a patient left home? How much time has a patient spent away from home? What if a patient has not left home all week? In the agoraphobia interventions, the questions focused on how close a patient has been to the mall (or other crowded place); or how much has a patient managed to escape from the comfort or security zone? For gambling, key questions were: has a patient approached a gambling area? How long has a patient been there? How many times has a patient left?

As a final step (the right side of Figure 1) and to address RQ3—*which interrelated dimensions may best inform users for designing context-aware mobile apps to support mental health interventions*—we evaluated the resulting relationships between the dimensions (outcomes of RQ2) with the existing literature. The objective was to evaluate these relationships with the evidence and to summarize them as a set of considerations that can inform therapists in the process of designing context-aware mobile applications for successful mental health interventions.

## 3. Results

### 3.1. Participants

Ten meetings were conducted between June 2017 and July 2018 to get a better understanding of how geospatial concepts and related technology could make a real, social impact for technology-driven interventions in mental health. The number of participants per meeting was approximately 4–5 people, with half being psychologists and the other half being computer scientists. Minutes of the meetings were summarized and centralized in a public GitHub repository (https://github.com/cgranell/labpsitec-ideas).

Regarding the focus groups, the first focus group engaged participants from multidisciplinary profiles (therapists, psychologists, computer scientists, architects) in a discussion about the relationships between context and mental health. In a first step, 12 professionals, three from each area, were contacted through phone calls and emails. Nine participants (*N* = 9) were finally recruited. Sociodemographic data of the participants is shown in Table 1. The focus group meeting took place 31 January 2017, in the Universitat Jaume I of Castellón (Spain), and lasted two hours.

The second brought together psychologists and therapists (eight people) involved directly with mental health interventions to discuss the relationships, benefits, barriers, and implications of mobile technology and mental health. We emailed initially 12 people from this group. Finally, eight participants (*N* = 8) were recruited and took part in the focus group meeting. Sociodemographic data of the participants is shown in Table 2. The focus group meeting took place 22 January 2018, in the Universitat Jaume I of Castellón (Spain), and lasted one hour.

### 3.2. Relevant Dimensions of Each Area of Knowledge [RQ1]

**Figure 2:**
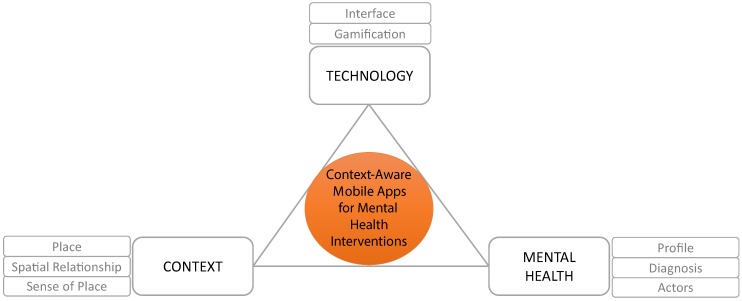
Areas of knowledge and the relevant dimensions that influence the design of context-aware mobile apps as mental health intervention.

In this section, we address RQ1—*what are the relevant dimensions that sustain or describe each area of knowledge?* Technology, mental health and context are the three areas of knowledge in which our exploration is framed (see Figure 2). However, as these areas cover a lot of ground, we reduce them, through document analysis in combination with other qualitative research methods (Figure 1), to a set of relevant dimensions. This reduction (not simplification) is necessary to identify the relevant aspects of the areas of knowledge that can make an impact on mental health interventions.

#### 3.2.1. Technology

Technology has had a long and successful marriage with psychological interventions for mental health, not only in the creation of new technology-based interventions and treatments, but also to streamline traditional health processes (e.g., sharing health records). Initial telemedicine approaches consisted of offering traditional therapist/patient interaction, possibly automated, through telephone calls or text messages [42]. With the arrival and the rapid proliferation and penetration of the smartphone (currently, 2.53 billion people use smartphones [43]), and the increase in functionality in the past years (e.g., connectivity, processing capacity and memory, detection capabilities), the range of mobile applications for mental health interventions is rapidly expanding (e.g., [44,45]). This rapid growth has opened a large number of possibilities in many areas of medical care but especially in mental health [22,46]. If mobile devices, technology and mobile computing have dramatically impacted (mental) health to the point of coining the term m-health [18], the use of video game technology as an innovative way to treat psychological treatments is also a reality [47]. These two ideas framed our exploration to identify key dimensions derived from the area of knowledge pertinent to technology.

With respect to mobile devices and mobile computing, we have witnessed an explosion of mobile application developments over the past few years. A quick review of the related literature tells us that the most widely used strategy has been the digitization of standardized questionnaires previously established in the mental health field in a mobile application, such as the patient health questionnaire (PHQ-9) for depression screening [48]. The momentary and ecological characteristics of these types of intervention are ensured thanks to the ubiquitous and pervasive nature of mobile devices [49]: patients can fill in, smoothly integrate during their daily routines, digital questionnaires (or other data collection techniques) at any time, anywhere. Beyond that, the hardware and software possibilities offered by these intelligent devices are, in most cases, underused. This suggests that most of these application developments are limited to moving from a traditional way of operating to a digital medium (i.e., digital transformation [50]), but paying little or no attention to the new characteristics of the digital medium [51]. For example, technical characteristics such as built-in sensors, location analysis, compatible operating systems, battery requirements, performance, network needs and communication interfaces are characteristics to be carefully evaluated to integrate them into intervention tools for the efficient delivery of content to patients. In this study, we group all these characteristics under the umbrella of the term "interface". Therefore, **interface** is one of the relevant dimensions of the technology area.

One of the main problems of mobile interventions is the lack of patient adherence to their treatment. A 2013 study identified engagement techniques as one of the essential features for the success of mobile interventions for mental health [52]. Videogames are a promising technology for improved participation and engagement practices [53]. Indeed, applications of online and offline video games for mental health therapies have been extensively reviewed [47,54], suggesting that different genres of existing video games could yield great benefits in mental health interventions. Rather than bringing existing video games to scenarios other than pure entertainment, our focus here is on how game design strategies and game dynamics can be applied to non-game contexts such as mental health. This is widely known as gamification [55]. The intention to use gamification in mental health interventions is to exploit its inherent ability to immerse a player (patient) in a story (intervention) to convey a key message and/or achieve a goal. For example, metaphors can help therapists to design an engaging game story for an intervention, such as Spallazzo and Mariani’s [56] location-based mobile game that turned the symptoms of depression and their consequences into a metaphorical story to improve the learning experience and increase the awareness of healthy people (players) with mental health illnesses. Other examples of gamified interventions for mental health are already being used [57]. Thus, it is worth exploring **gamification** as a relevant dimension related to compliance, engagement, and attractiveness to mitigate drop-out rates for interventions based on mobile devices.

#### 3.2.2. Context

The sedentary lifestyle is reaching alarming levels, which has not only a direct impact on cardiovascular health [58], but also a significant deterioration in mental health [59,60]. Several studies have shown that the distribution of the urban environment is an influential factor in the amount of mobility that a population presents, being therefore vital to address the issues due to a sedentary lifestyle. It is widely recognized that different features of the urban environment facilitate or complicate moderate physical activity [11,12] and has effects on mental health [14]. In our previous work, Miralles et al. [61] explored the natural and built environment features that were perceived as the most relevant ones to encourage physical activity. Features such as access to public open spaces, availability of bike lanes or presence of drinking fountains appear at the top of the list, in concordance with other studies [62,63].

The analysis of urban characteristics is an important factor when planning psychological interventions in cities, because people (patients) develop their daily routines and activities in their cities. Urban places are not only important because of physical proximity, but also because of the social and emotional bonds that people acquire in places that are meaningful to them. These two ideas drove our exploration of well-established theories in the fields of Geographic Information Science [64,65], Human Geography [66] and Environmental Psychology [16], to identify the relevant dimensions that relate to the urban context.

Ahlqvist and Schlieder [67] completed an in-depth analysis in the realm of geogames or location-aware games [68] to align existing theories of core geographic concepts in the GIScience field, namely Kuhn [69], Kuhn and Ballatore [70], and Janelle and Goodchild [71], to location-based game patterns such as exploring, collecting objects, co-locality, and spatial structure [72]. The authors propose a unified set of core geographic concepts and compare them to existing spatial game patterns [72], which resulted in two main groups of spatial patterns: locality and proximity. Due to their generality, we focus on them here and reinterpret beyond the geogames domain. Locality means to be in a place but accounting also for the topological relationships (e.g., disjoint, meets,) between geographic objects [73,74]. For example, the *meet* relationship means that two spatial regions are touched such as the boundary of the countries of Spain and Portugal, and the *disjoint* refers to separate regions such as each island in the Canary Islands. Considering these relationships, locality refers to entering a place, leaving it, or crossing it, all of them seen as triggered events [67]. Since locality implies a formal and informal way of specifying a place, we use **place** instead of locality because the former is intuitive and widely used in Human Geography and Environmental Psychology. Proximity, the second fundamental spatial pattern, is defined as *“the variation of distance to a neighborhood or location”* ([67], p. 9). We refer to proximity as the distance to a place (e.g., 500 m) or qualitative appreciation (e.g., close, far), considering it as a type of spatiotemporal association. Lü et al. [75] recently suggested the term relationship as *“a general term for spatiotemporal association among geographic elements” (p. 352)*. Built upon the notions of proximity, spatiotemporal association, and relationship, we propose **spatial relationship** as a relevant dimension of context.

People have emotional bonds to places. We perceive our surroundings according to our mood, feelings, and emotions. Places can evoke feelings and/or lived experiences [76,77], and are central pieces of human experience with implications for the development of identity, affection and empathy [78,79,80]. Place can be defined as *“a particular space which is covered with meanings and values by the users”* ([81], p. 187) and plays a significant role in human behavior and mental health [16]. For example, the way in which individuals perceive themselves depends on how they feel in their daily places and interact with them [82]. In this sense, the concept of sense of place has been studied for a long time in Environmental Psychology and Human Geography to refer to human experiences, emotions, thoughts, meanings, values and feelings associated with places [76]. Jorgensen and Stedman [83] define it as the cognitive and behavioral dimensions of the relationship that an individual has towards a specific geographic area. In Section 3.2.1, Lü et al. [75] also pointed out the concept of “relationship” and expanded it to cover *"other interactive linkage among multiple geographical elements"* in addition to spatiotemporal associations such as physical, chemical, and biological interactions. Built upon the concepts of sense of place, emotional bonds and places, we propose the **sense of place** dimension as a type of relationship between people and spatial settings.

#### 3.2.3. Mental Health

Although there are multiple approaches that address mental health, it is well known that Cognitive Behavioral Therapy (CBT) is one of the most dominant forms of psychotherapy [84]. Within the area of mental health, we intend to offer some reflections on the dimensions that should be considered when carrying out context-aware mobile apps interventions in the CBT approach.

To take into account the profile of the patient, who suffers a mental health issue, is key in designing an intervention. Recently, some aspects such as gender or age have been highlighted as important features to defining user preferences in technology-based psychological interventions and, specifically, in mobile applications for health [85,86]. Given this evidence, the first dimension proposed is the **profile** of the patient.

When a patient suffers from a specific disorder, it is important to establish the corresponding diagnosis and, consequently, the evidence-based treatments [87]. This may also imply the identification of the commonalities among the emotional disorders supported by the emerging extensions and innovations of the CBT approach, such as the unified transdiagnostic protocol [88,89]. The therapy will have requirements, including technical developments, which may imply great repercussions on cost and time. A close link between the logical therapeutic components for the target disorder and the technical requirements to increase efficiency rates will be thus necessary [45]. Therefore, we take **diagnosis** as the second key dimension.

It is common for family members, couples or people close to the patients to actively participate in the therapy too [90,91]. The term *actor* in software modelling specifies a role played by an external user or entity that interact with a system. Here, we borrow this terminology to refer to actors as therapists and familiar members, who are externals to the intervention but at the same time play a specific role in it. Therefore, it is important to take into account this possible variation of actors or other people in the interaction with the treatment itself, because it can have influence the technical developments that are applied in the intervention [91]. In consequence, the dimension of **actor** represents key roles besides the patient (e.g., therapists, caregivers, family members, etc.) involved in the therapeutic process. In summary, three key dimensions stem from the mental health area: the profile of the primary users or patients, the diagnosis, and the actors, encompassing other roles in the intervention.

A best practice in the document analysis method is to establish the meaning and contribution of the analyzed sources to the research topic [37]. To this end, Table 3 summarizes the main sources and references from the literature used for the re-interpretation of each identified dimension of our exploration.

### 3.3. Relations between Dimensions [RQ2]

**Figure 3:**
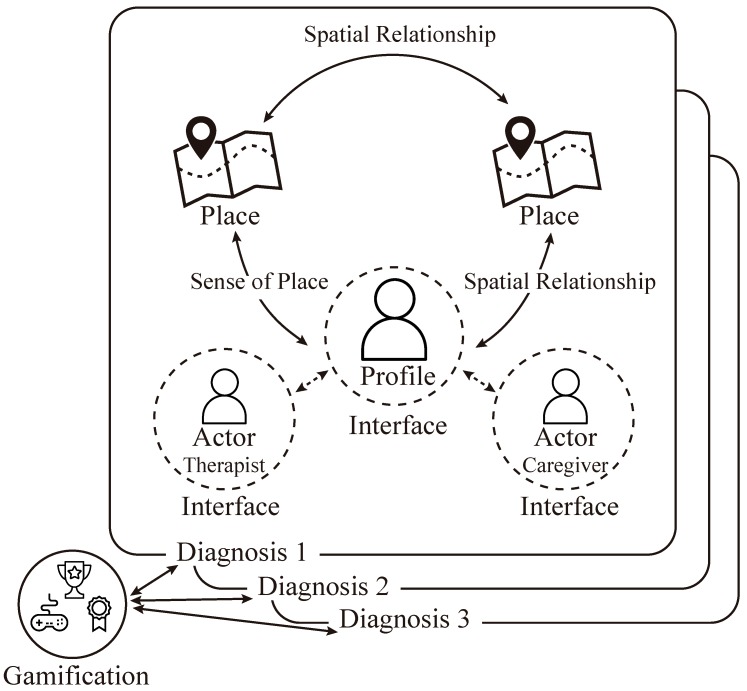
Relationships between dimensions. The diagnosis dimension drives the remaining dimensions, since different diagnoses bring different ways of organizing the other dimensions to design context-aware mobile applications as intervention tools.

Once the salient dimensions of the three areas of knowledge have been carefully selected, in this section we address RQ2—*how do dimensions belonging to distinct areas relate to each other?* We ran focus groups to explore the potential relationships between the identified dimensions (Figure 3) in the process of designing context-aware mobile apps for mental health interventions. The exploration is organized into sections, each of them covering a couple of areas at the same time, in the same way that the guided discussion was carried out during the focus groups.

#### 3.3.1. Technology and Context

The two dimensions within the context area respond essentially to the question of *where?*. This question is inevitable in the case of context-aware applications deployed in outdoors, urban environments. When observing the link between the dimensions, the relationship between place and spatial relationship emerges naturally. Places can also be visited in a particular order, establishing a temporal relationship between places. In addition to spatial and temporal associations, social and emotional bonds to certain places are especially relevant for mental health interventions. For example, the sense of place dimension can categorize places according to the feeling and emotion perceived by patients, producing places of “emotion”, places of “anxiety”, places of “fear”, etc. These types of relations between places (spatial, temporal and sense of place) permit us to capture relevant meanings and associations to places, transforming them into personal and unique places. It is a powerful mechanism for therapists and psychologists to specify places with meaningful roles for patients within a psychological intervention. Through the focus groups, we discovered that virtually all places have the three types of relationships, not necessarily equally balanced, because each of these relationships can be weighted differently for each place. The point was that these three relationships were rarely considered together in the design of psychological interventions. Although the spatial relationship between places (proximity, etc.) is an objective observation, it has not yet been considered as a key design variable in psychological interventions, a fact widely corroborated in the literature (Section 1).

Various factors may explain the above. First, the assumption that a place has a spatial connotation is widely accepted, but the problem arises on how to spatialize a place, i.e., how to define the precise spatial boundaries of a place attending to the sense of place [79]. Some authors even claim that the spatial representation of a place remains an open issue for the advancement both in Environmental Psychology [76] and GIScience ([96], p. 1). Second, some places may be selected by therapists for the emotional links they produce with patients, such as places that produce panic and places that should be avoided due to gambling addiction. However, these places are usually seen under the dominant relationship, whether spatial, temporal or sense of place (emotional), neglecting the role of other relationships with that place. Consequently, the two dimensions of the context are largely unexplored, especially when considered together.

The interplay between place and interface is well researched in some specific fields such as location-based services [97]. The communication and provision of timely, contextual information depending on the location of the mobile device and/or user, is a defining characteristic of location-based applications, which undoubtedly should be an integral part of mobile psychological interventions. To this respect, some research works have deployed easy-to-implement features, such as letting patients respond to questions, offering textual information and watching media content via mobile devices [98,99]. However, much attention must be paid to the development of more natural and non-intrusive location-based interfaces. This is clear, for example, for the delivery of personalized content and/or to take actions according to the distance (near/far) to a place, how much time a patient spends in a place, how long it takes to go from one place to another, or how often you visit a place, just to mention some situations. A patient can approach, enter, cross, stay in, and leave a place and this triggers an action that is made tangible through an interface. Although the distance travelled by a patient is not usually a measure or variable required in the treatments (except those related to physical activity), a participant of the focus group explicitly mentioned that the identification of special places *"is very interesting for addiction, in the sense of detecting ’dangerous’ places of a patient"*. The same participant added that *"the possibility of providing different levels of activation and/or alarms [when a patient is in these special places] is a really relevant and useful feature for the interventions"*. These are the type of interactions that can be developed when the dimensions of places, the spatial relationship, the sense of place and the interface are considered together. Therefore, the potential combinations between these dimensions are countless, and they must be carefully thought about for the intervention at hand, also considering the profile dimension, since any triggered action is directed, i.e., is customized, to a profile (i.e., a patient) through an interface.

The interrelation between place and gamification is also mediated by the spatial relationship and the sense of place. Sharma et al. [100] wondered how game designers select physical spaces to create places. In their review, they developed a detailed classification for the use of space in mixed reality games, which can also be understood according to the spatial (and temporal) relationship and the sense of place. In summary, the re-examination of the dimensions of technology and context allows therapists to walk through unexplored routes to discover new options for the design of mobile tools for mental health interventions.

#### 3.3.2. Context and Mental Health

Based on the premise that mental health has an enormous connection with physical activity, the results of Sallis et al. [11] suggest a direct, positive correlation between the elements of the urban context and physical activity. These relate immediately to the areas of context and mental health. In the realm of psychological treatments, capturing, understanding, and managing context, to better assess and intervene, is the ultimate goal. In general, the importance of the context was well recognized by the participants of both focus groups. The discussion turned to more general considerations relative to context such as, for example, what makes a place safe or, at least, a place that patients consider or perceive as safe. Safety can be seen from different points of view such as traffic, pollution, deterioration of buildings, and dirty streets, being a major problem in modern cities and, therefore, in the development of interventions in urban areas. Again, a good understanding of the structure and organization of the city or urban area where the intervention is planned to be developed is vital. The selected places for the intervention must be carefully considered. As one participant aptly summarized *“if patients feel the app is going to help, they will accept it”*. An app forcing patients to go to unsafe places may produce undesired effects on patients.

Participants also casted doubts about which features of the context were more relevant for each intervention. A variety of ways to collect contextual information exists, i.e., using sensors and analysis of the obtained data, using location-based services, communicating with peer devices in the surrounding environment, or asking the user [101]. Multiple examples from the literature demonstrate the degree of penetration and use of sensors in mobile devices for data collection, analysis, and context determination. That, of course, also applies to psychological treatments that are provided to people in real time during their everyday lives and in natural settings, anywhere [49]. In depression, for example, context-based techniques to recommend activities have been proposed [102,103]. Other examples capture the location information of patients to detect whether they are in a remote place, so that reminders or suggestions are sent accordingly [104]. The focus of Simm et al. [105] is on anxiety, and the authors use contextual information to allow patients to relate visually, through a mobile app, places to symptoms, linking them to the sense of place dimension. Finally, Skillen et al. [106] address caregivers (actors) related to patients with autism so that they can establish areas (linking again to the place and spatial relationship dimensions) where notifications are sent when the patient enters or leaves them.

#### 3.3.3. Mental Health and Technology

Gamification research, seen as a collection of game features, game elements, and mechanics that aim to motivate and engage users [92], involves a wide arsenal of theoretical approaches and practical actions and techniques [93]. Here we are not interested in any game scenario in general, but in mental health interventions in particular. Therefore, the following question focuses on the connections between gamification and profiles: What gamified characteristics can make interventions more attractive to patients (players)? To answer it an approach may be to analyze and segment target users to select the game elements and gamification techniques that better suit the needs of each user segment. In his seminal study, Bartle [94] associated the main characteristics of video games to user profiles, to create general categories of players such as the “explorer”, “socializer” or “achiever”. For example, asocializer player is especially interested in interacting with other players, and an achiever is focused on acting on the world. More interestingly, Bartle [94] established the path to enable connections between user profiles and game elements. Along this line, Tondello et al. [95] recently carried out an empirical exploratory research of the preferences of players in games, which resulted in nine game elements and five game styles associated with certain user profiles.

The below examples analyze games and player profiles together. These results do not imply that all game strategies and elements should be completely used for a user profile; it is simply advised to take them into account if a positive impact is expected on the effectiveness of the gaming applications. Yet, the aim of these examples can be extrapolated to mental health scenarios where adherence to use of a mobile device as part of a treatment is a priority. Mandryk and Birk [107] study the potential impact of game-based interventions on mental health, paying attention to the relationship between game elements and self-reported indicators of wellbeing by players (patients). The results suggest that games are a motivating attraction and provide considerable appeal, regardless of the age and gender of the participants, which shows that gamification is not limited only to young people, but prevails in all demographic groups.

**Figure 4:**
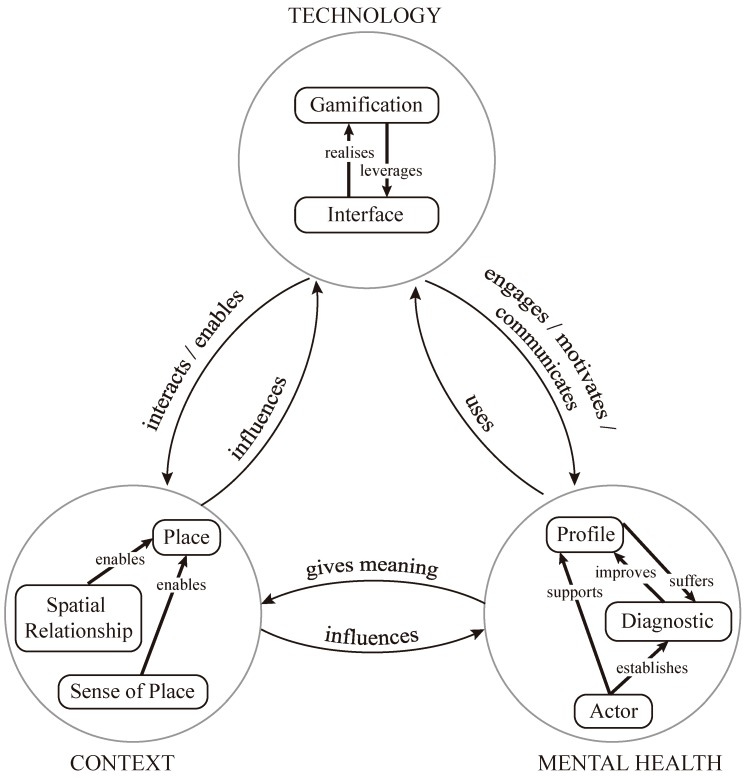
The design process of context-aware mobile apps is influenced by several interrelated dimensions that need to be considered as a whole.

Figure 4 summarizes the results of the RQ1 (identification of relevant dimensions) and RQ2 (meaningful relationships among dimensions). It gives an overview of the relationships between the dimensions and aspects that affect the design of context-aware mobile apps for mental health interventions, which will be discussed further in the following section.

## 4. Discussion

In this section, we address RQ3—*which interrelated dimensions may best inform users for designing context-aware mobile apps to support mental health intervention tools?* We discuss the interrelation between the aforementioned dimensions and provide some considerations screened against the literature, when possible, as well as limitations of the present exploration.

### 4.1. Considerations [RQ3]

“There is an app for that!” This is the intriguing title of a recent study by Van Ameringen et al. [45], in which the authors conducted a comprehensive literature review of the current state of mental health apps for anxiety, depression and mood disorders. Despite the unquestionable potential benefits of mobile apps for mental health assessment and treatments, the authors concluded that *“[o]verall there is a significant disconnect between app developers, the scientific community and health care, leaving the utility of existing apps questionable”* ([45], p. 526). The authors refer mainly to the multidisciplinary nature of the m-health field, in which research teams with different backgrounds and skills are more necessary than ever. Therefore, a fluid communication of scientific, technical, social, and psychological information between team members and patients is necessary. A good deal of research has shown the feasibility and benefits of the application of participatory and human-centered design in m-health [105,108].

We agree with the previous statements and argue that the design, implementation and delivery of context-aware mobile apps for mental health intervention are by nature a multidisciplinary and multidimensional field. Nevertheless, and despite the increasing use of participatory design methodologies, it seems clear that other aspects and factors can make a mobile application fail as an intervention tool. While we realize that its success depends on the combination of exogenous and endogenous variables, our message here is that a solid foundation to *connect and relate* the relevant dimensions identified in this paper is vital to mitigate unnecessary failures during the design process and the subsequent implementation process of these mobile interventions. While we have noted that substantial research has been conducted in many of these dimensions, we have also identified that a more holistic approach for designing mobile interventions is a clear gap in the literature, which we attempt to fill here. In summary, the aim of this study is to make developers and psychologists aware of the relationships between interdisciplinary dimensions to alleviate the lack of knowledge and connection between the areas of knowledge of technology, context, and mental health. In the following, we discuss how the relationships represented in Figure 4 can lead to a set of considerations to help developers and therapists design mobile interventions for mental health wellbeing.

As stated previously, place matters. Designers, psychologists, geographers and urbanists should work together to carefully consider the multiple relationships to places when selecting and incorporating places in mental health interventions [100]. The creation and identification of places, which gather subjective perceptions of the interaction between people and the environment and place value on them, can have a great impact on the effectiveness of mental health interventions. The availability of methods for the spatialization of place and place-related concepts such as sense of place is gaining importance, and practical approaches to compute it are under way [109]. This will undoubtedly facilitate the task of establishing spatial boundaries of places to leverage the dimensions of spatial relationship and sense of place altogether to be enabled through interfaces.

As places are central, a logical consequence is that locality is crucial for the success of context-aware mobile apps. It is expected that an intervention will be personalized to the places where it is deployed. In other words, a context-aware mobile app that leverages on *local* places cannot be experienced anywhere, since the affordances and emotional bonds created and generated with these local places are specifically designed for the local urban configuration and the profiles of the patients. Much attention should be paid to the cultural, social, urban morphology, and demography differences between cities or urban areas. In addition, as patients are also stratified according to socioeconomic and cultural variables, The identification of places is not only based on the morphology, structure, and urban organization, but designers must also take into account the profile dimension to choose the right places.

The profile dimension uses and interacts with the interface, regarding the latter as an envelope of the sensing, communication and interaction capabilities of mobile applications or devices [110]. The interface performs or implements the gamification dimension. The combination of the dimensions of interface and gamification is critical to augment the potential of interfaces to deliver content in novel ways to profiles (patients) and/or motivate them for sustaining adherence and engagement over long periods of time. These two features are much sought after by actors (therapists). In other words, gamification strategies applied to mobile applications for mental health interventions can be considered as *“activators of social and health change”*, as experimentally observed by Spallazzo and Mariani [56]. The authors also pointed out that gamification can be considered as a practice capable of impacting both designers (or therapists) and players (or patients) to establish multifaceted relationships between them. Therefore, taking into account the dimensions proposed and carefully considering the best way to relate them, it seems to be a recipe for successfully designing specific interventions. The proposed holistic process provides a wide range of means and strategies to carry out the chosen psychotherapy approach to influence patients and change their behaviors in the directions determined by the therapists.

### 4.2. Limitations

We have discussed here that the design of context-aware mobile apps for mental health interventions is affected and influenced by a reduced but comprehensive number of influential dimensions at a high level of abstraction such as place, spatial relationship, sense of place, interface, gamification, diagnosis, profile, and actors. As usual, the devil is in the detail. We have set aside much more detailed aspects and factors that must also be considered at later stages during the development process. For example, while the dimension gamification is appropriate at the time of design, specific game mechanics, strategies, and elements must be specified at some point in the development of a mobile intervention. The same is true for the dimensions of places and sense of places, which must be clearly specified during the development of the intervention. The key point is that all these dimensions must be carefully considered if context-aware mobile applications are used as intervention tools. Therefore, the dimensions, their relationships and design considerations do not allow designers, physiologists, and practitioners to *implement* a mobile intervention, but rather they intend to help them consider an integral interdisciplinary strategy when designing context-aware mobile apps for mental health interventions.

We also realize that a limitation of the paper is that the research approach adopted is eminently qualitative and, therefore, there is a lack of empirical data to validate our findings. On the positive side, however, we examined diverse sources through the document analysis method, along with other qualitative research methods (Section 2). This approach proved to be effective in practice to verify information from multiple documents and research papers, and helped us to order and reinterpret sometimes contradicting statements in the analyzed documents. The lack of empirical data will be addressed in future work as we are planning a series of experiments to test distinct mobile interventions for mental health diagnoses taking into account the dimensions and design considerations presented in this study.

## 5. Conclusions

Through a methodological approach that combines several qualitative research methods, we have re-examined key documents to identify a set of relevant dimensions of the three areas of knowledge (Technology, Context, and Mental Health). Next, we have re-interpreted the potential relationships between these dimensions, both within the same area of knowledge and between different areas. Finally, we have provided a set of considerations and validated them against existing literature. When therapists and developers strive to design and create mobile apps for mental health interventions, it is advisable that they take into consideration our findings for designing custom mobile applications for mental health interventions. Adhering to the holistic strategy described here can increase the possibilities of designing mobile interventions that keep patients motivated and engaged and, therefore, increasing the effectiveness of the psychological treatment.

Wykes et al. [7] argued that funding research on e-health and technology applied to mental health interventions *“generates a good return on investment, reducing not only the burdens on individuals and families but also the costs of care and support in the long term”* ([7], p. 2). Our intention here is to attract the attention of developers and therapists to comprehensively consider the multiple interactions between the relevant dimensions of all areas of knowledge, to capture physical and social context. Instead of concentrating and trusting only a few, they need to expand and make the diversity of dimensions and their relationships a strategy to discover new options for the design of context-aware mobile tools for mental health interventions.

## Figures and Tables

**Table 1:** Sociodemographic data of the participants of the focus group one.

Participant	Expertise/Professional Profile	Education Level	Gender	Age
Participant 1	Physical Education	Bachelor’s Degree	Male	35
Participant 2	Computer Science and Geographic Information Systems	PhD	Male	40
Participant 3	Clinical Psychology	PhD	Female	37
Participant 4	Psychology and Ergonomics	PhD	Female	40
Participant 5	Clinical Psychology	PhD	Female	44
Participant 6	Architect	PhD	Male	45
Participant 7	Physical Education	Bachelor’s Degree	Male	35
Participant 8	Computer Science and Education	PhD	Female	51
Participant 9	Computer Science and Education	PhD	Female	49

**Table 2:** Sociodemographic data of the participants of the focus group two. The ICTs Knowledge column represents the participant’s ability with the use of information and communication technologies. Zero means non-ICT knowledge and four denotes an expert level.

Participant	ICT Knowledge	Education Level	Gender	Age
Participant 1	2	PhD	Female	38
Participant 2	2	PhD	Female	30
Participant 3	3	Bachelor’s Degree	Female	35
Participant 4	2	Bachelor’s Degree	Female	26
Participant 5	2	Bachelor’s Degree	Female	31
Participant 6	4	PhD	Male	32
Participant 7	2	Bachelor’s Degree	Male	23
Participant 8	2	Bachelor’s Degree	Female	26

**Table 3:** Summary of selected dimensions grouped by area of knowledge, together with the main sources and references analyzed to reinterpret each dimension.

Area	Dimension	Main Sources and References
**Technology**	Interface	BinDhim et al. [48], Rana et al. [17], Haggerty [50], and Bohnsack et al. [51].
	Gamification	Seaborn and Fels [92], Nacke and Deterding [93], Bartle [94],
		and Tondello et al. [95].
**Context**	Place	Kuhn [69], Kuhn and Ballatore [70], Janelle and Goodchild [71],
		Ahlqvist and Schlieder [67], and Sintoris [72].
	Spatial	Ahlqvist and Schlieder [67], Lü et al. [75], Randell et al. [73], and
	Relationship	Egenhofer et al. [74].
	Sense of Place	Stedman [76], Tuan [77], Duff [78], Najafi and Shariff [81],
		Gotham and Brumley [82], Jorgensen and Stedman [83], and
		Acedo et al. [79].
**Mental Health**	Profile	Andone et al. [85], and Zeng et al. [86].
	Diagnosis	Nathan and Gorman [87], Van Ameringen et al. [45], Barlow et al. [88],
		and Ellard et al. [89].
	Actors	Turkington et al. [90], and Echeburúa et al. [91].

## Abbreviations

The following abbreviations are used in this manuscript:

CBT	Cognitive Behavioral Therapy
GPS	Global Positioning System
LABPSITEC	Laboratory of Psychology and Technology
PHQ	Patient Health Questionnaire
RQ	Research Question
WHO	World Health Organization
